# Are all “research fields” equal? Rethinking practice for the use of data from crowdsourcing market places

**DOI:** 10.3758/s13428-016-0789-y

**Published:** 2016-08-11

**Authors:** Ilka H. Gleibs

**Affiliations:** Department of Psychological and Behavioural Science, London School of Economics and Political Science, London, UK

**Keywords:** Ethics, MTurk, Crowdsourcing, Methodology, Sampling

## Abstract

New technologies like large-scale social media sites (e.g., Facebook and Twitter) and crowdsourcing services (e.g., Amazon Mechanical Turk, Crowdflower, Clickworker) are impacting social science research and providing many new and interesting avenues for research. The use of these new technologies for research has not been without challenges, and a recently published psychological study on Facebook has led to a widespread discussion of the ethics of conducting large-scale experiments online. Surprisingly little has been said about the ethics of conducting research using commercial crowdsourcing marketplaces. In this article, I focus on the question of which ethical questions are raised by data collection with crowdsourcing tools. I briefly draw on the implications of Internet research more generally, and then focus on the specific challenges that research with crowdsourcing tools faces. I identify *fair pay* and the related issue of *respect for autonomy*, as well as problems with the *power dynamic* between researcher and participant, which has implications for *withdrawal without prejudice*, as the major ethical challenges of crowdsourced data. Furthermore, I wish to draw attention to how we can develop a “best practice” for researchers using crowdsourcing tools.

Treat your workers with respect and dignity. Workers are not numbers and statistics. Workers are not lab rats. Workers are people and should be treated with respect.

–turker “T,” a Turkopticon moderator

New technologies like large-scale social media sides (e.g., Facebook and Twitter) and crowdsourcing services (e.g., Amazon Mechanical Turk, Crowdflower, Clickworker) have made an impact on social science research and provide many new and interesting avenues for research (Bond et al., 2012; Paolacci, Chandler, & Ipeirotis, 2010). Moreover, crowdsourcing services have become increasingly important and popular tools for participant recruitment in the psychological sciences and beyond. For many social scientists in the USA and elsewhere, Amazon’s Mechanical Turk^1^ (MTurk) is a primary source for crowdsourced research, and some empirical studies in top journals of our field have been largely based on data collected on MTurk (e.g., Baldwin, Biernat, & Landau, 2015; Greenaway et al., 2015; Heintzelman, Trent, & King, 2013; Hui, Chen, Leung, & Berry, 2015). In addition, the current “replication crisis” in (social) psychology (Earp & Trafimow, 2015) highlights the need for larger samples and “easy” access to diverse participants. Thus, the use of crowdsourcing marketplaces for data collections seems to play an important part in increasing access to large enough samples and to overcoming the overreliance on undergraduate students (Barchard & Williams, 2008; Behrend, Sharek, Meade, & Wiebe, 2011; Buhrmester, Kwang, & Gosling, 2011; Dandurand, Shultz, & Onishi, 2008; Horton, Rand, & Zeckhauser, 2011; van Steenbergen & Bocanegra, 2015).

Despite the great amount of literature that has dealt with online data collection and its benefits and difficulties (Buhrmester et al., 2011; Litman, Robinson, & Abberbock, 2016; Litman, Robinson, & Rosenzweig, 2015), less (especially in the psychological literature) has been said about the ethics of conducting research using commercial crowdsourcing markets (but see Busarovs, 2013; Chandler & Shapiro, 2016; Fort, Adda, & Cohen, 2011; Irani & Silberman, 2013; Goodman & Paolacci, manuscript submitted for publication). However, in the last year there have been several media stories about the life and working conditions of “Turkers,” and with that, the ethics of conducting research in “science factories” (e.g., Dholakia, 2015; Marder & Fritz, 2015). In addition to these critical media stories, recent efforts from crowdworkers themselves have highlighted some poor academic practices on MTurk (Salehi et al., 2015). Taken together, these accounts demonstrate that an uncritical use of these research sites and the assumption that MTurk and other platforms elicit the same types of ethical concerns as more traditional forms of recruitment and data collection (Barchard & Williams, 2008; Shank, 2016) might have to be questioned.

In the present article, I argue that our ethics must be developed in response to the development and application of new sampling methods. In particular, we need to focus on the specific social contexts of MTurk and of workers on MTurk. That is, when we discuss the use of MTurk (and other platforms) for data collection, we should not only focus on data quality and the validity of the obtained results, but also on how workers are treated as participants and the relationship between researchers (requesters) and participants (workers).

## The rise of MTurk as a research tool

Despite initial concerns about the use of MTurk and other tools, researchers have now embraced the use of crowdsourcing marketplaces for data collection, with their prospect of immediate access to a large and diverse participant pool (Horton et al., 2011; Landers & Behrend, 2015). Some researchers see MTurk as an important tool that has democratized science^2^ by providing easier and cheaper access to research participants (Kraut et al., 2004; Paolacci & Chandler, 2014). This might be especially the case for researchers from smaller institutions or less senior researchers, who do not have access to large labs on campus and/or sufficient participant pools. In addition, this emerging research field helps reduce the overreliance on undergraduate students as participants (Aguinis & Lawal, 2012; Behrend et al., 2011; Gosling, Sandy, John, & Potter, 2010; Henrich, Heine, & Norenzayan, 2010; Stewart et al., 2015).

Notwithstanding the many benefits of crowdsourcing as a research tool, there have been discussions of the limitations of MTurk and other sources. These have predominantly focused around data quality and issues of sample composition, representativeness, and the attention span and nonnaïveté of MTurk workers (Hauser & Schwarz, 2015; Landers & Behrend, 2015; Paolacci et al., 2010; Roulin, 2015). Whereas some people have raised doubts about the reliability and validity of data (the *Journal of Vocational Behavior*, for example, does not accept papers based on paid online panels), others have argued that the data quality is often (but not always) comparable to that of more traditionally sourced data (Buhrmester et al., 2011; Casler, Bickel, & Hackett, 2013; Hauser & Schwarz, 2015; Roulin, 2015). Thus, much of the work in this domain is concerned with how to increase participants’ performance and the data quality from crowdsource marketplaces. For example, there is now evidence that most crowdsourced data are of higher quality than (or are just as good as) data collected from more “traditional” samples. For example, Crump and colleagues (2013) showed that a number of important experiments from cognitive psychology, including the Stoop, flanker, and Simon paradigms, could be replicated using MTurk samples. Similarly, Casler and colleagues (2013) compared participants recruited from MTurk, social media, and a university setting, and could not find differences in performance on an object selection and categorization task. Generally, it is thought that data quality on MTurk is good and mostly reliable (Paolacci & Chandler, 2014; D. N. Shapiro, Chandler, & Mueller, 2013; Weinberg, Freese, & McElhattan, 2014).

Less research has focused on the perception of the MTurk workers as research participants and the specifics of MTurk as a field of research (Irani & Silberman, 2013; Salehi et al., 2015; Silberman & Irani, 2016; Silberman, Irani, & Ross, 2010). Yet, issues regarding the privacy of workers, workers’ rights and appropriate compensation, and—more generally—the well-being and protection of participants from crowdsourced marketplaces are pertinent, and are emerging as issues in law (Felstiner, 2011) and other disciplines (Bederson & Quinn, 2011; Brawley & Pury, 2016; Wolfson & Lease, 2011). Following on from these accounts, I will argue that we have to focus on the specific ethical challenges that arise when researchers collect data with MTurk. In particular, we should focus on the specifics of this field of research and the interaction between researchers and MTurk workers. Therefore, in the remainder of this article, I review the benefits of crowdsourced research, briefly describe the challenges and implications of Internet research more generally, and then focus on the specific challenges that research with crowdsourcing tools faces. As a further point, I will draw attention to how we can develop “best practices” for researchers using crowdsourcing tools.

## Benefits of crowdsourced research

Modern psychology has a long tradition of relying on undergraduate students as a main source for data collection. Some authors (Baumeister & Bushman, 2011) have argued that psychologists do not need to recruit from other populations, because “[social] psychology is mainly interested in normal, typical people, as opposed to unusual groups . . . (e.g., children or mentally ill people),” and “College students are drawn from a broad segment of normal people” (p. 20), making them ideal participants. However, others have (maybe more convincingly) argued that psychology is overly relying on Western, educated, industrialized, rich, and democratic (WEIRD) people (Gosling & Mason, 2015; Henrich et al., 2010; Sears, 1986), who are not representative of most people in the world. Following this line, psychologists and other social scientists increasingly include more diverse samples in their research.

Thus, the first advantage is that MTurk and other crowdsourcing marketplaces offer an exciting opportunity to (possibly) recruit from more representative and diverse populations than simply undergraduate students (Barchard & Williams, 2008; Behrend et al., 2011; Buhrmester et al., 2011; Dandurand et al., 2008; Horton et al., 2011). More specifically, the majority of MTurk workers come from the USA (68.7 %), and most of the remainder from India (29.31 %; Ipeirotis, 2010). The US sample from MTurk is typically older (their average age is 36 years) and has more work experience than undergraduate students. In total, MTurk workers tend to be younger, overeducated, underemployed, less religious, and more liberal than the general US population (Berinsky, Huber, & Lenz, 2012). Hence, MTurk’s participant pool of workers is large and diverse, but it should not be treated as a representative sample. Still, it is an important source to complement and substitute for other convenience samples that psychologists have traditionally relied on (Paolacci & Chandler, 2014; Roulin, 2015).

The second major advantage of crowdsourced data collection is its relatively inexpensive and rapid nature, which makes it much easier to recruit sample sizes that bear sufficient statistical power for psychological experiments (Goodman, Cryder, & Cheema, 2013; Rand, 2012; Suri & Watts, 2011). Especially in light of recent discussions about underpowered studies in psychology (Earp & Trafimow, 2015; van Steenbergen & Bocanegra, 2015), the recruitment of large sample sizes is becoming increasingly vital (Crump, McDonnell, & Gureckis, 2013). With MTurk and other services, researchers can usually recruit several hundred participants within hours or days. Stewart and colleagues (2015) estimated that one (behavioural science) laboratory could recruit about 7300 online participants in each quarter of a year.

Thus, using crowdsource marketplaces is now an important part of sourcing data, and it highlights how modern psychological research greatly benefits from the possibilities that the Internet provides.

## Ethical dilemmas in Internet research

Since the late 1990s, the Internet has been used in psychological research, both as a field of study and a field in which we study (Gosling & Bonnenburg, 1998; Kraut et al., 2004; Postmes, Spears, & Lea, 1998; Sassenberg, 2002). However, adoption of this new technology was relatively slow, and in 2003–2004 Skitka and Sargis (2006; cited in Gosling & Mason, 2015) identified only 22 studies, out of 1,401 published in journals of the American Psychological Association (APA), that made use of the Internet. Today psychologists have wholeheartedly embraced Internet research, and thousands of studies now collect their data via the Internet. Moreover, the Internet is not just used for data collection, but is in itself a research field that generates new and previously unavailable opportunities for studying social interaction, offering the chance to observe new behavior and human experiences (Buchanan & Zimmer, 2012).

However, developments in Internet research also pose new ethical challenges (Fiske & Hauser, 2014; Kahn, Vayena, & Mastroianni, 2014). Several of these challenges have been identified and addressed in recent years, and they tend to focus on issues such as informed consent, debriefing, and which data are public or private, as well as on issues of anonymity (Bruder, Göritz, Reips, & Gebhard, 2015; Buchanan & Williams, 2010; Gleibs, 2014; Gosling & Mason, 2015; Kahn et al., 2014; Sabou et al. 2012).

One ethical challenge that is especially pertinent to research using the Internet, and the case of crowdsourced data collection in particular, is whether there is a difference between commercial and public use of data collected via third parties such as MTurk (Vayena, Salathé, Madoff, & Brownstein, 2015). Whereas some argue that the growing reliance on public–private collaboration will become increasingly integral for large-scale social science, and that this alliance will lead to better-regulated research guidelines in both the private and public realms (Kahn et al., 2014), others warn that the private sector is charging ahead and creating de facto standards for data use that provide broad access to personal information and behavior (R. B. Shapiro & Ossorio, 2013). The question of commercial parties and the introduction of market forces in research participation is one that is essential for understanding the ethical consequences of crowdsourced research, which is done mostly via commercial crowdsourced marketplaces such as MTurk.

## Ethical dilemmas in crowdsourced research

Psychological studies that are conducted on MTurk and other crowdsourcing platforms undergo the same ethical reviews as other lab or field studies do, and the use of crowdsourced data is usually not questioned. This is done under the assumption that the ethical challenges associated with research on MTurk should not be different from those faced in other “fields” (Barchard & Williams, 2008; Shank, 2016); in essence, research environments are treated fairly similarly, irrespective of whether data are collected online or offline. Yet, I argue that crucial differences between the old and new means of collecting data pose ethical challenges, and I focus on two: namely, the issue of *fair pay*, which is linked to the ethical principle of respect for autonomy, as well as the issue of *asymmetrical power relations*, which are related, for example, to problems with “withdrawal-without-prejudice” on crowdsourcing marketplaces.

Partaking in research on MTurk has become a means to increase or sustain an income for a sizeable number of people; essentially, these workers become “professional crowdworkers” (Fort et al., 2011; Martin et al. 2014; Silberman et al., 2010). This is in contrast to the typical psychology research participant, who is most likely a student or other volunteer, and who is compensated for time and/or expenses but does not expect a “wage.” To illustrate, many webpages and online discussion groups (e.g., reddit.com, pennyhoarder.com, Turker Nation) have discussed and explained how to make an income from MTurk. Moreover, Martin and colleagues (2014) showed in an ethno-methodological study that the main reasons for participation on MTurk is to earn money, and that participants describe MTurk as a labor market (Brawley & Pury, 2016; Jiang, Wagner, & Nardi 2015; Litman et al., 2015).

This is despite the fact that compensations are very low on MTurk, and individual researchers often give only small incentives^3^ (see also http://wearedynamo.org; Downs, Holbrook, Sheng, & Cranor 2010; Fort et al., 2011), which might give them the impression that participants take part because they are internally motivated (Buhrmester et al., 2011). However, we know that about 10 % of the US workers on MTurk report household incomes below $15,000, and 25 % below $25,000 per annum (for a real-time tracker of key demographics—i.e., age, gender, marital status, income, household size, and country—see the MTurk Tracker by Panos Ipeirotis, at http://demographics.mturk-tracker.com; see also Chandler & Shapiro, 2016; Ross, Irani, Silberman, Zaldivar, & Tomlinson 2010; Silberman et al., 2010). In addition, D. N. Shapiro et al. (2013) reported that 56.7 % of their sample was underemployed (8.6 %), unemployed by choice (14.7 %), or unemployed but preferred not to be (24.4 %); 20.1 % reported a household income of less than $20,000, and another 26.9 % an income below $40,000 (see also Chandler & Shapiro, 2016).

Given these data, we can assume that for some participants on MTurk (but clearly not all; see Chandler & Shapiro, 2016, who argued that fun and learning new skills are also important elements of worker motivation) the money earned there is an important source of income (Brawley & Pury, 2016). In addition, Litman and colleagues (2015) showed that the motivation of MTurk works has shifted from being primarily intrinsic (fun, learning new skills, or passing time with a purposeful activity) to being mainly driven by financial rewards, or an extrinsic motivation. To be more precise, whereas Buhrmester and colleagues (2011) found that many workers found participating in their study enjoyable and interesting and were mostly intrinsically motivated, more recent work has reported that monetary compensation is the primary motivator for participation among US- and India-based users (Brawley & Pury, 2016; Litman et al., 2015). Therefore, when participating on MTurk as a requester, we should be aware that individual human intelligence tasks (HITs; e.g., studies) can be part of a pool of tasks that accumulate to provide a low-paid service income. Even though the work by Litman et al. indicated that monetary compensations did not necessarily influence the data quality, we have to consider the ethical implications of making research participation a source of income, which are linked to the power differential created by an employer–contractor relationship and the question of whether workers can “afford” to reject tasks.

This needs to be understood in terms of the premise that one of the big advantages of MTurk is the relatively low pay. In addition, one could argue that it is a “free-market” that regulates which tasks or HITs are done and how much is paid for them (Buhrmester et al., 2011). Although wages are set at the requesters’ discretion, workers are free to do (or not do) a specific HIT. Thus, workers have the power to withdraw their contribution from the market. In addition, if wages became too high it would become too expensive to run an experiment on a crowdsourced platform; less work would then be available, and workers would struggle. Thus, wages that are too high could have detrimental effects for requesters and workers alike (Ipeirotis, 2010; Goodman & Paolacci, manuscript submitted for publication). Yet, on forums such as Turkopticon, one of the most common complaints of workers is the low pay, and even though a worker might want to stop doing tasks that underpay, he or she might find it difficult to switch to “better” requesters, because 98 % of the tasks are created by 10 % of the requesters (Kingsley, Gray, & Suri, 2015).

The issue of participant pay is not a great concern for most psychological research. Usually, participants are compensated for their time and efforts, and standard rates for paid participation in psychological/behavioral science research are between $8 and $10 per hour in the US, or about £10 in the UK (Goodman & Paolacci, manuscript submitted for publication). Yet, in biomedical research the question of payment and incentives is considered more controversial (Dickert & Grady, 1999; Grant & Sugarman, 2004; Ripley, 2006). For example, Dickert and Grady (1999) identified the concern that when participants’ payment becomes part of a regular income (which, in case of medical research, can be in excess of $1,000), it violates the ethical norms of the investigator–participant relations by turning it into a commercial relationship. Thus, the participants enter into a dependency relationship with the researcher, in which risks of violating basic ethical norms—such as not coercing participants, informed consent, and protection of autonomy—might increase (Grant & Sugarman, 2004). Ethics codes in both medicine and the social/behavioral sciences include the right to withdraw; but if participants are wage earners, are they still able to do so? According to Grant and Sugarman, this is a question that only arises when compensation comes in the form of a “wage” and when “participants” become contractors delivering a specific service or work for a certain wage (see also Anderson & Weijer, 2002, for a discussion of the research participant as a wage earner in medical research). Under these circumstances, participants might find it more difficult to renege on the contract between researchers and participants in order to withdraw from the study.

Following on the discussion of whether the workers on MTurk are not only research participants, but contractors or “wage earners,” some scholars have argued that MTurk has developed into an unregulated labor marketplace with very low wages, incomplete contracting, weak access to enforcement, and a disciplining role of reputation, in which workers are denied basic workplace rights and the community has no recourse for employer wrongdoing (Benson, Sojourner, & Umyarov, 2015; Felstiner, 2011; Fort et al., 2011). Interestingly, Amazon legally defines workers as contractors who are subject to laws designed for freelancers and consultants; in this way, they get around paying workers a minimum wage (Irani & Silberman, 2013).

In essence, the requester (and therefore the researcher) engages in an employer–contractor relationship that has shifted away from the common relationship between investigators and participants, as a revenue-neutral experience, to one in which the requester is a client and the participant a contractor. In addition, requesters hold more power than the workers in setting wages and withdrawing work. This, in turn, can violate one important pillar of human participant research—namely, the *respect for autonomy*, which implies that we must protect the rights, freedom, and dignity of our participants (Rosnow, 1997).

Another example of how this problem might manifest is the fact that requesters (researchers) can reject work by workers, who consequently will not be paid for the HIT. Silberman and Irani (2016) have described the complications of the rejection feature very well, explaining how the reject feature gives the requester unique power over the workers and increases the workers’ uncertainty. Rejecting a HIT can occur because the worker did not fit the study criteria or did not do the work correctly, but also because of completion code malfunctions. When an employer rejects work, the worker’s approval rating goes down. Approval rates, however, are essential, because to be eligible for a task, a high approval rate is expected from workers, and most requesters do not accept workers with an approval rate below 95 % (Peer, Vosgerau, & Acquisti, 2014). Rejections leave workers with a mark counting against them on their “permanent record” at MTurk, which may take them below the 95 % threshold. Importantly, the conditions regarding approval or rejection are not questionable by workers. Employers (requesters) can reject work without payment, which might lead to a decline in the worker’s approval rate, and no built-in system on MTurk is available to appeal rejections (Silberman & Irani, 2016). Thus, a rejected HIT is not only an immediate loss of income, but it can also impact future earnings, so rejections need to be clearly justified by researchers.

This process of rejection can be contrasted to what happens in research labs in the “physical” world. In an average behavioral research lab, participants are hardly ever “rejected” or expelled from the participant pool—for example, because of poor performance or a lack of attention. Also, in the “offline world” participants can receive prompt responses from researchers or lab managers, who are obliged to answer these requests and can help resolve issues regarding performance and so forth. Theoretically, workers can contact requesters through MTurk’s Web interface, and they should also be able to contact the institutional review board (IRB; or ethics committee) that approved a study. However, MTurk workers have no legal recourse against employers who reject work, and workers have only limited routes to voice dissent within MTurk itself.^4^ As per Amazon’s contract, the requester is not required to answer (Irani & Silberman, 2013), and IRBs have only limited power to sanction a researcher who does not pay appropriately or does not answer e-mails. In addition, when workers “return” a job (i.e., withdraw from a study) or want to challenge a study, this might negatively affects their reported completion rate. Whereas most (academic) requesters are well-intentioned, it is difficult for workers to distinguish between well-intended and less-well-intended requesters, and there are few costs for requesters who do not engage with workers (see Silberman & Irani, 2016, p. 12, for an example).

What these examples highlight is that MTurk is based on a certain power differential, which is related to a certain degree of information asymmetry and the anonymity of the Web. This is linked to the way that MTurk is designed as an “‘artificial’ artificial intelligence” device, in which requesters outsource tasks that are difficult to handle through machines alone (Chandler & Shapiro, 2016). Thus, it was not designed with the workers in mind or as a place in which people would actively participate in research activities.^5^ Therefore, on MTurk itself workers have very little information on the prospective requester, and only limited information on the task; they also have little opportunity to dissent with a specific requester in terms of pay, the nature of the task, and the task description (Benson et al., 2015; Felstiner, 2011; Irani & Silberman, 2013). Likewise, requesters know little about their participants. That is, they have almost no control over the conditions under which studies are completed, whether participants read and understood consent form, or whether they read debriefing materials (Gosling & Mason, 2015).

In summary, compensation and the consequences of approval rates have been identified as two particular ethical challenges. Workers on MTurk are paid relatively little and have no guaranteed payment, and withdrawal from a study (or HIT) has financial and reputational consequences. This might have implications for our understanding of “voluntary” research participation. In addition, it also might have an influence on demand effects by influencing behaviors that indicate being a “good participant” (Nichols & Maner, 2008). Again, this could be perceived as an advantage, because research has shown that high-reputation workers (i.e., those with approval rates above 95 %) are very good at attention checks and produce high-quality data (Peer et al., 2014).

However, it might also be related to the fact that workers who are new to MTurk (or, indeed, high-reputation workers) are motivated to avoid rejection rates, and are therefore unlikely to withdraw or be rejected (Goodman & Paolacci, manuscript submitted for publication). Thus, the inherent power differential and information asymmetry makes it difficult for participants to “freely” choose to participate in or leave studies. This might have consequences for whether the *“withdrawal-without-prejudice” principle* that is embedded in our ethical contracts (see Standard 8.02a; APA, 2010) is honored in research that makes use of MTurk. In this way, I argue that the ethical challenges stemming from online research using MTurk are important to address carefully when conducting research using crowdsourcing platforms.

## Addressing dilemmas when doing research on MTurk

How can we as social scientists address these issues? First, and foremost, we should understand MTurk workers (or other members of crowdsourcing platforms) not as “subjects” or anonymous workers who provide us with easily accessible data, but as active participants who make important contributions to our work and research in general. This is well demonstrated in the opening of this article, where an MTurk worker was quoted as saying: “Workers are not numbers and statistics. Workers are not lab rats. Workers are people and should be treated with respect.” Thus, the present article is intended to bring the topics of labor relations, fair pay, and the experiences of MTurk workers to the attention of researchers and to increase mindfulness about the workers’ experiences when conducting online research. This is in line with the notion that social scientists should strive to develop empathy for their research participants’ circumstances and the idea that the ethical issues that surround our research have to be taken into account when collecting data (Selznick, 2000). This also means that we should perceive participants as stakeholders in research and increase their agency in the process. With this perspective, we have to honor their valued contribution of time, attention, and cooperation, and acknowledge and reward these in a just and equitable way (Rosnow, 1997; Wright & Wright, 1999, 2002).

On a more practical level, we should *ensure fair pay* and adherence to ethical norms through self-management, but also in the publication process. This goes hand in hand with *greater transparency* when we provide information on samples. For example, journals could require authors to pay minimum-wage scale (from the respective participants’ country of residence) incomes to crowdsourced participants and to report the average length of a study for a better understanding of the pay per hour. In addition, universities/departments and research agencies that fund research should consistently provide funding that is adequate to pay participants at least a minimum wage. Moreover, IRBs that are responsible for human participant research should take into account the standards of fair pay and employment protection when making judgments on the ethical standards of studies. This is not to say that this is not done already, but just to highlight that the focus of increasing transparency is important not only to avoid questionable research practice in data analysis (John, Loewenstein, & Prelec, 2012), but also in relation to sampling methods.

Power differentials that influence the withdrawal-without-prejudice principle might be best addressed by ensuring that enough information on a particular study and when and how HITs are rejected by requesters are made public before MTurk workers start a study. This is analogous to what happens in the lab, where participants are informed about the study and the conditions of withdrawal. Furthermore, a short survey at the end of each study that asks about fair pay, perception of the study, and whether workers would recommend others to partake could be included (see Corti & Gillespie, 2015, for an example). This information should be made available with the publication of a study to ensure an ethical approach in conducting the research. In addition, a link could lead to the requester’s or study’s webpage (e.g., for preregistered studies, the link could lead to the preregistration information) for more information on the researcher and their work. Part of this goal has already been achieved by activist systems, such as Turker Nation and Turkopticon (Irani & Silberman, 2013; Silberman & Irani, 2016), that evaluate workers’ relationship with requesters and embed information about requesters (which was collected by workers) as part of the MTurk interface. Beyond that, more information on requesters could be made available by MTurk itself (similar to the approval rates of workers). In sum, with these measures, requesters would not only increase the transparency of their data collection approach, but also ensure *agency* for their workers. In essence, we should be more transparent in our use of MTurk and provide sufficient information for IRB bodies, in publications but also when conducting the research itself. Importantly, these recommendations are in line with Guidelines for Academic Requesters that were developed by a group of academics and by MTurk workers themselves (http://wiki.wearedynamo.org/index.php?title=Guidelines_for_Academic_Requesters&printable=yes), and I suggest that academic researchers should actively endorse and adhere to these guidelines.

In addition to solutions on an individual level, more collective approaches could be useful. First of all, attention could be drawn to alternatives to MTurk. Crowdsourcing platforms do exist that are created by academics and are dedicated to the sole purpose of doing research (see, e.g., Prolific Academic), and these might be viable alternatives to commercial platforms, which are not necessarily designed to fulfill the requirements of academic research. In addition, MTurk has led to several third-party solutions that can be practical means to increase researchers’ interaction with the participant pool. One example is *TurkPrime* (Litman et al., 2016), which allows researchers to target specific samples within the MTurk population, but also automates the approval process, enhances communication with participants, and monitors dropout and engagement rates.

A much broader and long-term solution would be the establishment of a national or international nonprofit infrastructure for online research. Bruder and colleagues (2015) proposed the construction of such a nonprofit, online research infrastructure that would provide access to a large (possibly representative) participant pool, integrated experiment/survey software, educational materials, and a data archive. This infrastructure should be available for qualified scientific users, similar to access to scientific libraries or archives. Such an initiative would also counteract an overreliance on commercial providers such as MTurk (the recent hike in commission costs at MTurk illustrates another vulnerability of reliance on commercial providers), and it would ease and assure the discipline-wide implementation of ethical guidelines. Moreover, such a noncommercial (inter)national online lab would also be in a better position to ensure data protection and data archiving that would be in line with the discipline’s ethical guidelines and legal requirements (Bruder et al., 2015). Of course, the question is who would bear the costs and practical management of such an infrastructure? Moreover, who would be able to share information on such an online platform (thus, would this be a national or international, a uni- or multidisciplinary, endeavor)? These questions remain open and make a wider discussion necessary.

## Conclusion

The purpose of this article has been to highlight some of the emerging challenges of engaging in online research and the use of crowdsourced marketplaces like MTurk for data collection. In the age of the “sharing economy” (Belk, 2014a, 2014b; Eckhardt & Bardhi, 2015) and “big data” (Mayer-Schönberger & Cukier, 2013), hopes are high for getting new insights by observing social interactions on a larger scale than can usually be reached with offline sources. In this article I have highlighted the fact that MTurk elicits different ethical concerns than other research environments, and I argued that when we discuss the use of MTurk and other platforms for data collection, we should not only focus on data quality and the validity of the obtained results, but also on issues of working conditions and fair pay, and how users are treated when doing research with us. Thus, it is our responsibility as scholars to ensure that our research methods and processes remain rooted in longstanding ethical practices. I argued that offline and online research environments are not entirely equivalent and that crowdsourcing platforms warrant special attention. Hence, the issues of fair pay, withdrawal without prejudice, and a commitment to participants as active agents and stakeholders in research become more pressing and have to be discussed in their context when dealing with MTurk and other commercial providers of large online panels.

The lack of an ethical concern with these issues can hinder academic progress, our regard as a community, and our trustworthiness as academics. We ultimately need an earnest, innovative, and creative discussion in the field as to how to implement ethical guidelines that first and foremost will protect participants, but also will allow researchers to conduct sound research. I propose that we start to reconsider the social contract of ethical do’s and don’ts between researchers and participants. To do this, we have to engage in a discussion on the ethical issues of conducting research on MTurk, in order to ensure that we treat participants as stakeholders in research and not as passive objects or merely a human resource (Trinidad et al., 2011). Researchers, funders, and IRBs must reconsider the issues of fair pay and engagement with research participants on crowdsourced marketplaces, as well as the challenges provided by Internet research. As a field, we should make sure that our work has social value that promises knowledge creation but also respects research participants, and that we are on the front line of setting standards for accessing and working with online sources that are in line with our ethical consciousness and research practice.

## Footnotes

1 Amazon Mechanical Turk is not the only crowdsourcing platform that is used for academic research. Others, such as SurveyMonkey, Qualtrics, and Prolific Academic, pay substantially higher rates; for example, Prolific Academic, a UK-based platform, endorses ethical reward that compensates participants with at least £5 ($7.50) per hour, or services like Qualtrics or SurveyMonkey charge $5–$10 for the completion of a 10-min survey. In the present article, I mainly focus on MTurk, as it is to date the most widely used crowdsourcing platform for academic research (Chandler & Shapiro, 2016).

2 This, however, is only true for US American researchers and their collaborators, because MTurk requires that requesters provide a US billing address and a credit card, debit card, Amazon Payments account, or US bank account in order to publish HITs (https://requester.AMT.com/help/faq#do_support_outside_us, page visited August 26, 2015). Thus, researchers from outside the US only have access to MTurk via a US American colleague who provides the necessary credentials.

3 To illustrate this point, I searched for human intelligence tasks (HITs) that contained the keyword “psychology” on April 29, 2016 (http://tinyurl.com/h5q8whq), to get a better understanding of the rewards paid on MTurk for research-related tasks. In all, 116 HITs were available, of which 53 were independent studies (several HITs were part of the same study or were follow-up studies). The average reward for these HITs was $0.68 (*N* = 58; *SD* = 0.45, range = $0.04–$4.00); not all studies indicated the time that was required, but on the basis of those that did (*n* = 31), we calculated that the average length for a study was 12.70 min (*SD* = 29.69, range = 1–50 min), which would indicate that the average wage would be $3.40/h, One of the top requesters for behavioral research (the Stanford GSB Behavioral Lab) pays about $0.1 per minute, which adds up to $6.00/h (from March 30 to April 29, 2016, the Stanford GSB Behavioral Lab did about 2,195 HITs and paid $1,129.40 in rewards ($0.52/HIT).

4 But see Turkopticon, a system that coordinates peer support, to hold requesters accountable by providing reviews about requesters, and places a button next to the requester on the MTurk window (https://turkopticon.ucsd.edu). Here the requester is rated by workers on four dimensions (communicativity, generosity, fairness, and promptness), on a scale from 1 to 5; bad reviews (ratings under 3) let workers avoid “bad” employers, and good reviews can help them find fair and honest requesters.

5 Academic research (and in particular, psychological research) represents a fraction of the work that is done on MTurk. To our knowledge, no reliable data make explicit how many HITs on MTurk are research-related, because Amazon does not publish detailed usage statistics. However, the Stanford GSB Behavioral Lab is among the top requesters (see the MTurk tracker: www.MTurk-tracker.com/#/toprequesters), and a PBS Newshour piece on “The Internet’s Hidden Science Factory” (Mader & Fritz, 2015) reported on a worker who estimated that she had completed more than 20,000 academic surveys in the last five years (see also DeSoto, 2016). In addition, Rand et al. (2014) estimated that the median MTurk worker had completed 300 academic studies (20 of which had occurred in the past week; the median number of total MTurk jobs was 1,500), as compared to less than ten studies done by participants from the Harvard Decision Sciences Lab (Rand et al., 2014). Given this information, we can conclude that sizeable amounts of the work on MTurk (on the basis of Rand’s data, around 20%) are research-related tasks.
